# Integrative Analysis Revealing Human Adipose-Specific Genes and Consolidating Obesity Loci

**DOI:** 10.1038/s41598-019-39582-8

**Published:** 2019-02-28

**Authors:** Jinsoo Ahn, Huiguang Wu, Kichoon Lee

**Affiliations:** 1 Department of Animal Sciences, The Ohio State University, Columbus, OH 43210 USA; 2 College of Veterinary Medicine, Yangzhou University, Yangzhou, 225009 China

## Abstract

Identification of adipose-specific genes has contributed to an understanding of mechanisms underlying adipocyte development and obesity. Herein, our analyses of the recent Genotype-Tissue Expression (GTEx) database revealed 38 adipose-specific/enhanced protein coding genes, among which 3 genes were novel adipose-specific, and 414 highly differentially expressed genes (DEGs) between subcutaneous and omental adipose depots. By integrative analyses of genome-wide association studies (GWASs), 14 adipose-specific/enhanced genes and 60 DEGs were found to be associated with obesity-related traits and diseases, consolidating evidence for contribution of these genes to the regional fat distribution and obesity phenotypes. In addition, expression of *HOXC* cluster was up-regulated in subcutaneous adipose tissue, and the majority of the *HOXB* cluster was expressed highly in omental adipose tissue, indicating differential expression patterns of *HOX* clusters in adipose depots. Our findings on the distinct gene expression profiles in adipose tissue and their relation to obesity provide an important foundation for future functional biological studies and therapeutic targets in obesity and associated diseases.

## Introduction

Obesity is one of the major global health issues because of its relation to various metabolic complications including type 2 diabetes, coronary heart disease, hypertension, dyslipidemia and a number of cancers^1–4^. Previous studies regarding obesity identified a significant contribution of genetic factors to obesity traits^5,6^. Among genetic factors, genes prominently expressed in adipose tissue are involved in various metabolic and endocrine functions of adipose tissue such as adipocyte development, lipid metabolism, glucose homeostasis and immune/inflammatory responses^7–9^. Altered expression of these adipose-specific genes leads to an increased release of fatty acids, hormones, and pro-inflammatory cytokines that contribute to obesity-related metabolic diseases^10^.

Functional studies about adipose-specific genes have increased our understanding of adipocyte biology and their etiological significance for the obesity and related diseases. These adipose-specific genes include genes encoding LEP/leptin (adipokine)^11,12^, ADIPOQ/adiponectin (adipokine)^13,14^, peroxisome proliferator-activated receptor gamma (PPARγ; adipose-specific transcription factor)^15–17^, and fatty acid binding protein 4 (FABP4; adipocyte fatty acid binding protein)^18,19^. In the early 2000s, high throughput screening methods including gene filter and gene chip arrays became available. Several groups, including ours, identified adipose-specific genes including *ADSF*/resistin^20^ and *ATGL*/desnutrin^21^. Our group has also identified several adipose-specific genes (e.g., *ISG12b1* and *ACVR1C*) based on the relative level of gene expression through mining bioinformatics databases^9,22^. To rigorously screen additional novel adipose-specific genes in humans, we have used the most recent Genotype-Tissue Expression (GTEx) data that provide up-to-date RNA-Seq transcriptomic profiling for various human tissues from hundreds of postmortem donors which is a suitable resource for identifying tissue-specific genes across multiple tissues^23,24^.

A large number of candidate genes for obesity have been documented by genome-wide association studies (GWAS) to determine genetic factors associated with obesity^25–29^. Despite findings from these studies, evidence linking adipose-specific genes and obesity in humans is still unclear. The primary objectives of this study were to identify novel adipose-specific genes and consolidate candidate genes for obesity-susceptibility by integrating GWAS data. In addition, depot-related expression of *HOX* genes in subcutaneous and visceral (omental) adipose tissues was examined to comprehensively evaluate developmental gene expression patterns for regional fat distribution.

Herein, 3 novel common adipose-specific genes and 414 differentially expressed genes (DEGs) between subcutaneous and omental adipose depots were identified. By integrating data of GWAS, evidence of interrelationships between those genes and major obesity-related traits or diseases including adiposity, type 2 diabetes, blood lipids, inflammation, and waist-to-hip ratio, were solidified. Furthermore, differential expression patterns of *HOX* genes in different adipose tissue depots were identified. Overall, our analysis of diverse databases have identified novel adipose-specific genes and consolidated evidence for their genetic relationship with obesity, providing a basis for further elucidation of therapeutic targets for obesity and related diseases.

## Results

### Identification of adipose-specific genes

**Figure 1:**
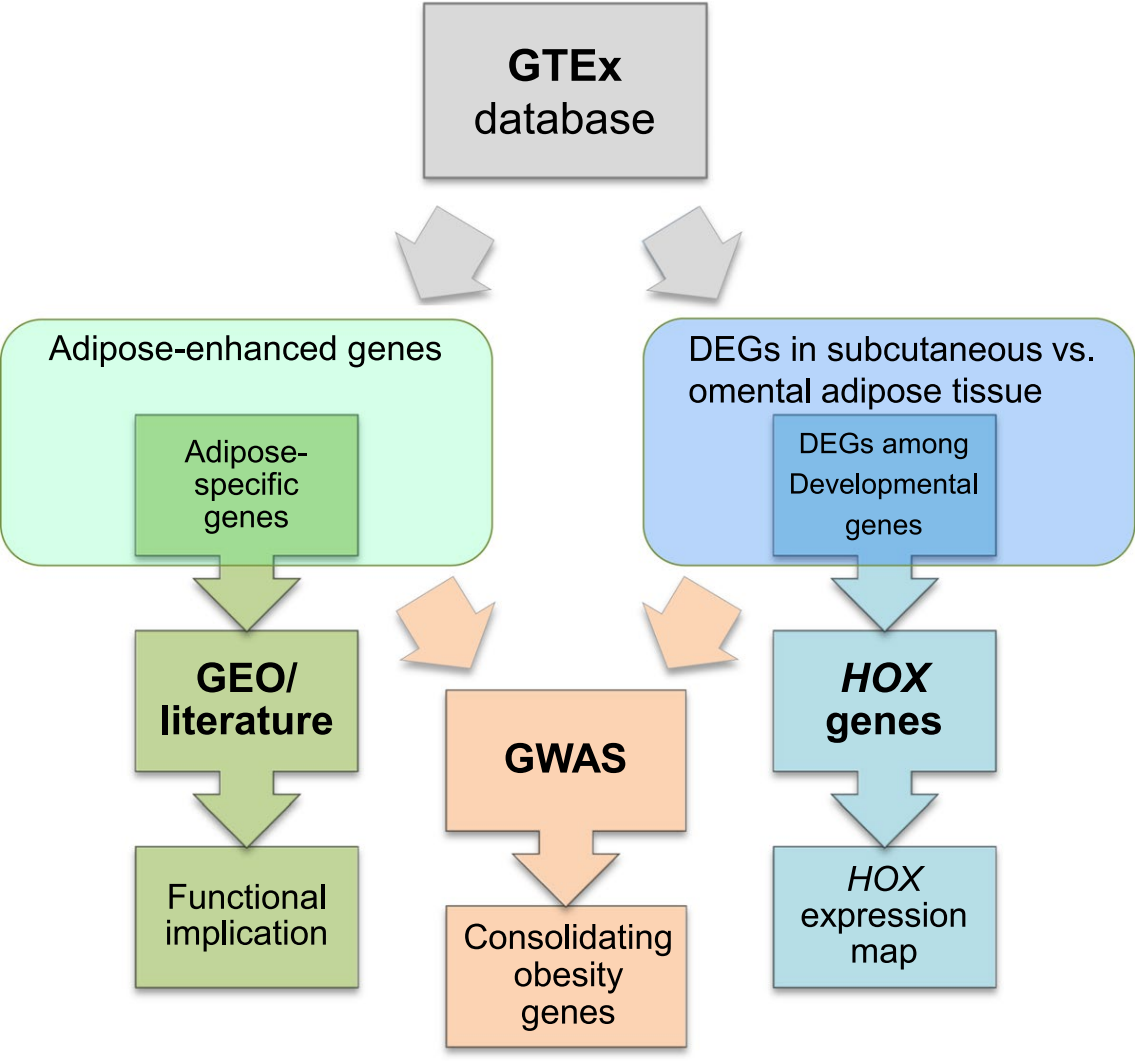
Work flow to identify adipose-related genes in humans, which were used to consolidate obesity-associated genes. Gene TPMs across 46 human tissues were collected from the GTEx v7 study, followed by data processing in three ways: (1) newly identified adipose-specific genes were investigated using GEO database and literature; (2) adipose depot-related DEGs of developmental genes were analyzed to construct a depot-based *HOX* expression map; and, (3) both adipose-enhanced genes and depot DEGs were located to mapped obesity-related loci published in GWAS.

**Figure 2:**
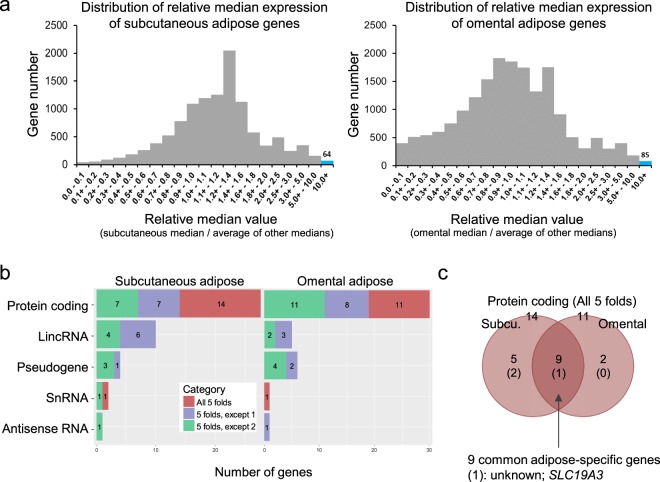
Classification of genes highly expressed in in human adipose tissues. (**a**) Distribution of adipose-enhanced genes, in which a median expression value in adipose tissue is more than 10-fold higher compared to an average of median expression values from all other tissues. (**b**) Adipose-specific genes whose median expression is more than 5-fold higher in adipose tissue compared to all other tissues (All 5 folds), and other adipose-enhanced genes whose median expression is more than 5-fold higher in adipose tissue compared to all other tissues, except one tissue (5 folds, except 1) and two tissues (5 folds, except 2). The significance of differences was set at a threshold of FDR-adjusted *P* value < 0.01. (**c**) Venn diagram of subcutaneous- and omental-specific genes. Genes whose expression is specific to both subcutaneous and omental adipose tissues are indicated in the intersection (n = 9), among which one gene, *SLC19A3*, is functionally unreported in adipose tissue.

**Figure 3:**
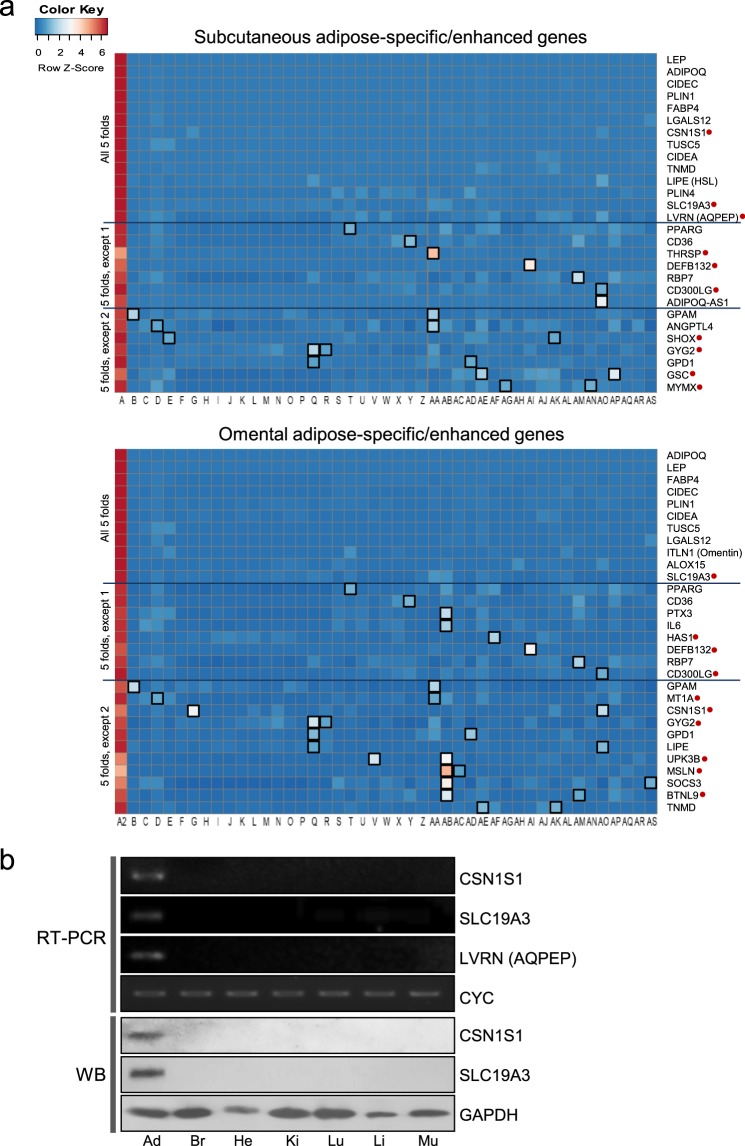
Expression of adipose-specific genes and adipose-enhanced genes. (**a**) Heat map of the expression of adipose-specific genes (all 5 folds) and adipose-enhanced genes (5 folds, except 1 and 5 folds, except 2) in various tissues [A: subcutaneous adipose tissue, A2: omental adipose tissue, and B through AS: 44 non-adipose tissues (B: Adrenal Gland, C: Artery - Aorta, D: Artery - Coronary, E: Artery - Tibial, F: Brain - Amygdala, G: Brain - Anterior cingulate cortex (BA24), H: Brain - Caudate (basal ganglia), I: Brain - Cerebellar Hemisphere, J: Brain - Cerebellum, K: Brain - Cortex, L: Brain - Frontal Cortex (BA9), M: Brain - Hippocampus, N: Brain - Hypothalamus, O: Brain - Nucleus accumbens (basal ganglia), P: Brain - Putamen (basal ganglia), Q: Brain - Spinal cord (cervical c-1), R: Brain - Substantia nigra, S: Colon - Sigmoid, T: Colon - Transverse, U: Esophagus - Gastroesophageal Junction, V: Esophagus - Mucosa, W: Esophagus - Muscularis, X2: Heart - Atrial Appendage, Y: Heart - Left Ventricle, Z: Kidney - Cortex, AA: Liver, AB: Lung, AC: Minor Salivary Gland, AD: Muscle - Skeletal, AE: Nerve - Tibial, AF: Ovary, AG: Pancreas, AH: Pituitary, AI: Prostate, AJ: Skin - Not Sun Exposed (Suprapubic), AK: Skin - Sun Exposed (Lower leg), AL: Small Intestine - Terminal Ileum, AM: Spleen, AN: Stomach, AO: Testis, AP: Thyroid, AQ: Uterus, AR: Vagina, AS: Whole Blood)]. Functionally unreported adipose-specific genes are indicated with red filled circles. Tissues with median fold changes less than five compared to adipose tissue are marked with black unfilled squares. (**b**) Semi-quantitative RT-PCR and/or Western blot analysis (WB) of *CSN1S1*, *SLC19A3*, and *LVRN* (*AQPEP*) in selected tissues: subcutaneous adipose tissue (Ad), brain (Br), heart (He), kidney (Ki), lung (Lu), liver (Li), and skeletal muscle (Mu). Target sizes for RT-PCR: 213 bp (*CSN1S1*), 170 bp (*SLC19A3*) and 193 bp (*LVRN* (*AQPEP*)). Expected sizes for full-length proteins were 21.7 kDa and 55.7 kDa for CSN1S1 and SLC19A3, respectively. Cyclophilin (*CYC*) (325 bp) and GAPDH (38 kDa) were used as a reference gene and protein, respectively.

Prior to initiating our workflow (Fig. 1), the GTEx dataset was downloaded from the GTEx portal (www.gtexportal.org), and then adipose-specific genes under the category of adipose-enhanced genes were explored. Distribution of medians in the GTEx dataset was first examined by plotting the number of genes against their relative median values, defined as a median expression value of subcutaneous or omental adipose tissue divided by an average of other medians (Fig. 2a). Most of the data were centered around the value 1 (indicating no difference), and fewer values on the right side of the value 1 represent adipose-enhanced expression (for example, expression of 64 subcutaneous adipose genes and 85 omental adipose genes were more than 10-fold). After the above initial evaluation of the dataset, adipose-specific genes were investigated under rigorous criteria of more than median-5-fold in all pairwise comparisons and an FDR-corrected *P* value < 0.01. As a result, 14 subcutaneous adipose- and 11 omental adipose-specific protein coding genes were identified (Fig. 2b; Supplementary Table 1). There were 9 genes that were overlapped between subcutaneous and omental tissues, and *SLC19A3* has not been reported in terms of its function in adipose tissue (Fig. 2c; Supplementary Table 1). Regarding subcutaneous exclusive expression, the functions of the *CSN1S1* gene and *LVRN* (also named as *AQPEP*) gene have not been reported, respectively. Heat maps and boxplots display expression levels of the adipose-specific genes in various tissues with extreme specificity to adipose tissue (Fig. 3a; Supplementary Figs 1–6; Supplementary Table 1). Semi-quantitative RT-PCR and/or Western blot analysis confirmed adipose-specific expression of *CSN1S1*, *SLC19A3*, and *LVRN* (*AQPEP*) (Fig. 3b; Supplementary Fig. 7). Adipose-enhanced genes with at least median-5-folds, with the exception of one or two pairwise comparisons, are also found and listed (Figs 2b and 3a; Supplementary Table 1).

### Comparative profiling of unreported adipose-specific genes

To obtain insight into roles of unreported adipose-specific genes in adipose tissue, functional annotations of the genes and information from literature describing their potential functions in other tissues and results from analysis of GSE data were summarized (Table 1). Among these adipose-specific genes, *CSN1S1* is involved in immune/inflammatory responses^30^, *SLC19A3* is responsible for thiamine transport and subsequent energy production^31^, and *LVRN* (*AQPEP*) is a member of the aminopeptidase family that catalyzes peptide catabolic processes^32^. Of particular interest, GSE data (GSE9624 and GSE12050) showed that *SLC19A3* and *CSN1S1* genes were expressed significantly higher in omental and subcutaneous adipose tissue, respectively, of an obese population than a lean population (Table 1). Given that those genes have more significant expression in fat cell fraction containing mature adipocytes than in stromal vascular fraction mainly containing preadipocytes and tendency of increasing expression during differentiation of human adipose-derived stem/stromal cells (Table 1), these three unreported adipose-specific genes may play important roles in adipogenensis and lipid metabolism and remain to be investigated.

**Table 1:** Comparative analysis of adipose-specific genes whose function in adipose tissue has not been reported, based on GEO Series (GSE) and literature search.

Gene	Relative median value	Location	General function	GSE80654	GSE9624 omental	GSE12050 subcutaneous	GSE53638 hASCs
				FC/SV	ob/lean	ob/lean	differentiation
*CSN1S1*	80.07 (s)	E	immunomodulatory properties^30^	8.32**	1.60	2.49**	—
*SLC19A3*	38.92 (s)/30.38 (o)	PM	thiamine transmembrane transport^31^	17.52***	2.16*	1.02	↑
*LVRN (AQPEP)*	26.85 (s)	PM	peptide catabolic process^32^	3.86***	0.78	1.15	↑

*LVRN* (*AQPEP*) is also known as FLJ90650. *s* subcutaneous; *o* omental; *E* extracellular; *PM* plasma membrane; *FC* fat cell; *SV* stromal-vascular; *ob* obese; *hASCs* human adipose-derived stem/stromal cells. *P < 0.05; **P < 0.01; and ***P < 0.001.

### GWAS associated phenotypes of adipose-specific/enhanced genes

The recent GWAS catalog of 71,423 records for 2,688 diseases or traits was used to select and classify 34 diseases or traits into four obesity-associated categories: obesity, type 2 diabetes, blood lipids, and inflammation (Supplementary Table 2). In order to identify obesity candidate genes among adipose-specific/enhanced genes, GWAS mapped and reported genes in those four categories were integrated with adipose-specific/enhanced genes. In total, 14 adipose-specific/enhanced genes were matched with those mapped and reported genes: obesity-related (4 genes), blood lipids-related (7 genes), type 2 diabetes-related (2 genes), and inflammation-related (4 genes) groups (Table 2). Among the 14 genes, 2, 5, and 7 genes were subcutaneous, omental, and common adipose-specific genes, respectively (Table 2; Supplementary Table 2). 13 genes have one-related GWAS catagories. Specifically, *PPARG* gene belongs to all four GWAS catagories (Table 2; Supplementary Table 2). Genes functionally non-reported in adipose tissue showed a link with a GWAS obesity trait (*SLC19A3* and *UPK3B* genes) and a GWAS blood lipids trait (*LVRN*, *CD300LG*, and *HAS1* genes) (Table 2). Linking of these adipose-specific/enhanced genes to those GWAS categories solidified evidence of their interrelationships with obesity and obesity-related traits.

**Table 2:** Adipose-specific/enhanced genes associated with four categories of GWAS phenotypes.

Gene	Region	Expression group	Specifically expressed tissue	Related trait category
*SLC19A3* ^*^	2q36.3	All 5 folds	Both	Obesity
*LVRN* (*AQPEP*)^*^	5q23.1		Subcutaneous fat	Blood Lipids
*LEP*	7q32.1		Both	Type 2 diabetes
*FABP4*	8q21.13		Both	Obesity
*ITLN1*	1q23.3		Omental fat	Inflammation
*ALOX15*	17p13.2		Omental fat	Inflammation
*PPARG*	3p25.2	5 folds, except 1	Both	Blood Lipids; Inflammation; Obesity; Type 2 diabetes
*CD36*	7q21.11		Both	Blood Lipids
*CD300LG* ^*^	17q21.31		Both	Blood Lipids
*IL6*	7p15.3		Omental fat	Inflammation
*HAS1* ^*^	19q13.41		Omental fat	Blood Lipids
*GPAM*	10q25.2	5 folds, except 2	Both	Blood Lipids
*ANGPTL4*	19p13.2		Subcutaneous fat	Blood Lipids
*UPK3B* ^*^	7q11.23		Omental fat	Obesity

^*^Genes that have not been reported regarding their function in adipose tissue.

### DEGs between adipose depots and associated obesity-related traits

**Figure 4:**
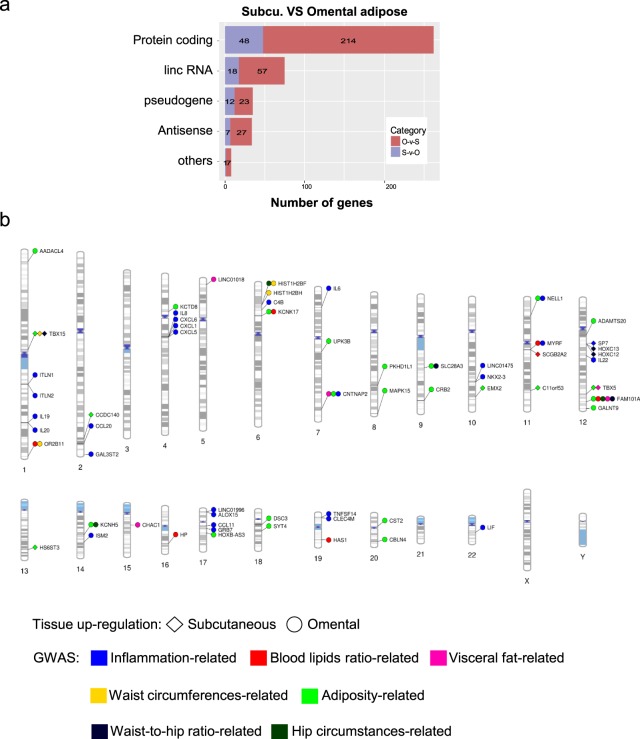
Classification and whole genome visualization of differentially expressed genes between two different adipose depots. (**a**) Classification of DEGs between subcutaneous and omental adipose tissues. DEGs in the two adipose depots were categorized into five groups including protein-coding, lincRNA, pseudogene, antisense RNA, and others. (**b**) Plotting DEGs between two different adipose tissues. The graphics illustrate association between DEGs in two different adipose depots and previously reported genes from GWAS studies plotted on ideograms of all chromosomes. Lines are plotted on chromosomal regions corresponding to the matched locations between DEGs and GWAS loci and then connected to different shapes representing different tissue abundancy: diamond, highly expressed genes in subcutaneous adipose tissue; circle, highly expressed genes in omental adipose tissue. GWAS phenotypes are color-coded to represent different categories. A detailed list of phenotypes and associations is presented in Supplementary Table 4.

DEGs between subcutaneous and omental adipose tissues were identified and then subjected to GWAS integration and KEGG pathway analyses to understand functional characteristics of those genes. A total of 414 DEGs (log2 fold change ≥3) were found, among which 86 and 328 DEGs showed up-regulated expression in subcutaneous and omental adipose tissue, respectively, and the protein-coding DEGs were 48 and 214 in subcutaneous and omental adipose tissues, respectively (Fig. 4a; Supplementary Table 3; Supplementary Fig. 8).

Using the recent GWAS catalog, 26 diseases or traits were grouped into seven relevant adipose depot-related categories (Supplementary Table 4). In order to find the evidence of a link between the adipose depot-related DEGs and GWAS locus, mapped and reported genes in those seven categories were integrated with DEGs listed in Supplementary Table 4. Among DEGs, a total of 60 genes were matched with GWAS mapped and reported genes in those seven categories with some overlaps: waist circumferences-related (3 genes), hip circumferences-related (3 genes), waist-to-hip ratio-related (5 genes), adiposity-related (25 genes), blood lipids-related (7 genes), inflammation-related (27 genes) and visceral fat-related (5 genes) (Fig. 4b; Supplementary Table 4). Among the 60 genes, 10 and 50 genes were up-regulated in subcutaneous and omental adipose tissue, respectively (Fig. 4b; Supplementary Table 4). Most inflammation-related GWAS genes (26 out of 27) were up-regulated in omental adipose tissue compared with subcutaneous adipose tissue, indicating omental adipose tissue plays a more important role in inflammation than subcutaneous adipose tissue. 48 and 8 DEGs have one and two related GWAS categories, respectively; and *TBX15* and *CNTNAP2* genes belong to three GWAS categories; and *FAM101A* gene is a candidate gene for five GWAS categories (Fig. 4b; Supplementary Table 4).

**Figure 5:**
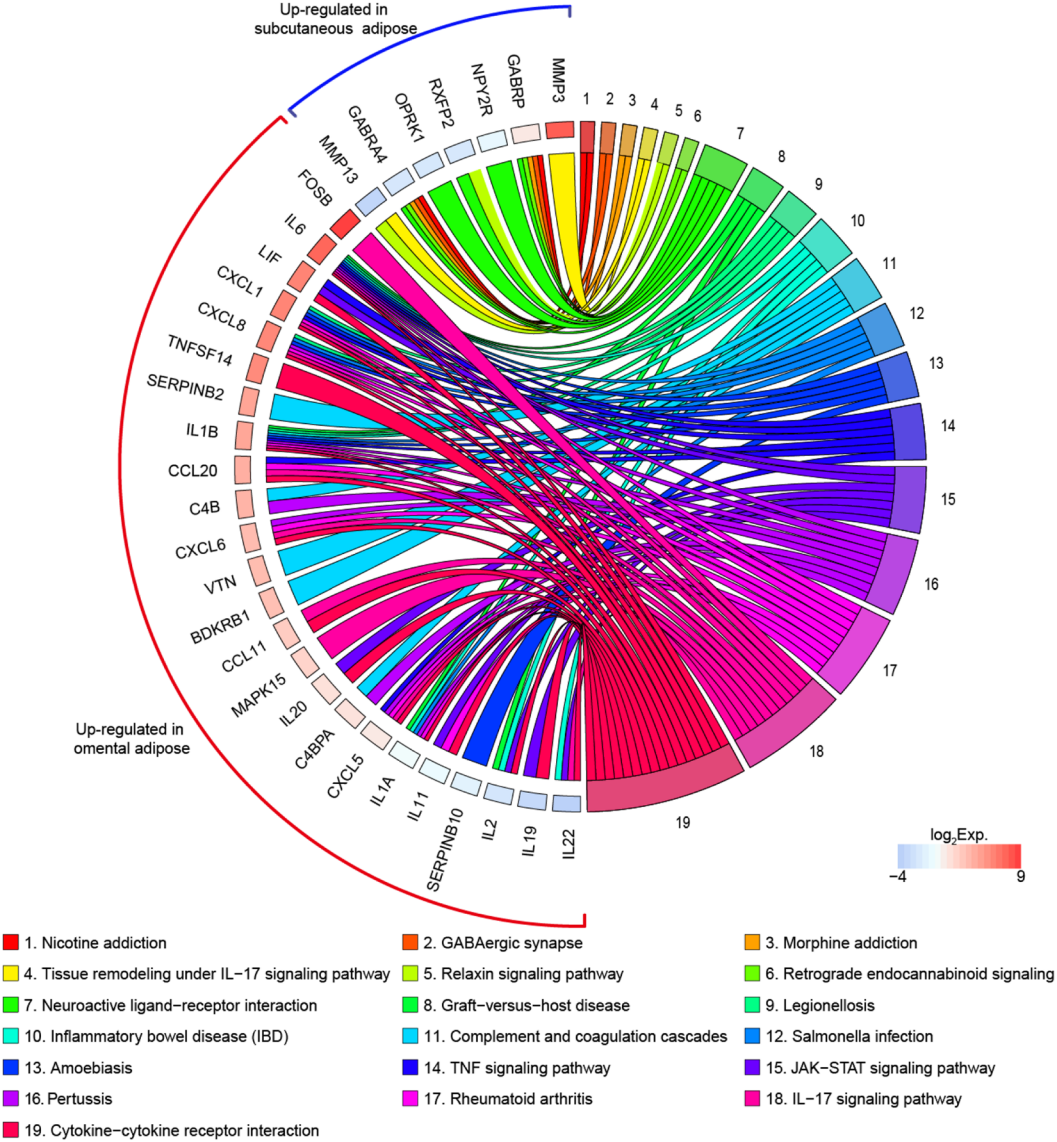
KEGG pathway analysis of up-regulated DEGs in subcutaneous and omental adipose tissue. Chords represent a detailed relationship between the expression levels of up-regulated DEGs (left semicircle perimeter) and their enriched KEGG pathways (right semicircle perimeter). For each gene, the expression value (TPM) of up-regulated DEGs in omental adipose tissue is shown by colored rectangles. Details in Supplementary Tables 5 and 6.

KEGG pathways analysis showed that up-regulated DEGs in subcutaneous adipose tissue were enriched in nicotine addiction, GABAergic synapse, morphine addiction, tissue remodeling, relaxin signaling pathway, retrograde endocannabinoid signaling, and neuroactive ligand-receptor interaction (Fig. 5: category 1 through 7; Supplementary Table 5). Further KEGG pathway analysis showed that significantly up-regulated DEGs in omental adipose tissue were highly related to categories including JAK-STAT signaling pathway, IL-17 signaling pathway, TNF signaling pathway, salmonella infection, amoebiasis, inflammatory bowel disease (IBD) and cytokine-cytokine receptor interaction, indicating those DEGs in omental adipose tissue might be involved in immune/inflammatory responses, pathological infection, and cell signaling. (Fig. 5: category 8 through 19; Supplementary Table 5).

Overall, many DEGs which showed distinct expression patterns between subcutaneous and omental adipose tissues were associated with adipose depot-related parameters such as waist-to-hip ratio and different enriched pathways, strengthening the evidence that those DEGs may be potential indicators of regional adipose tissue distribution.

### Dissecting expression patterns of *HOX* clusters in adipose tissues

**Figure 6:**
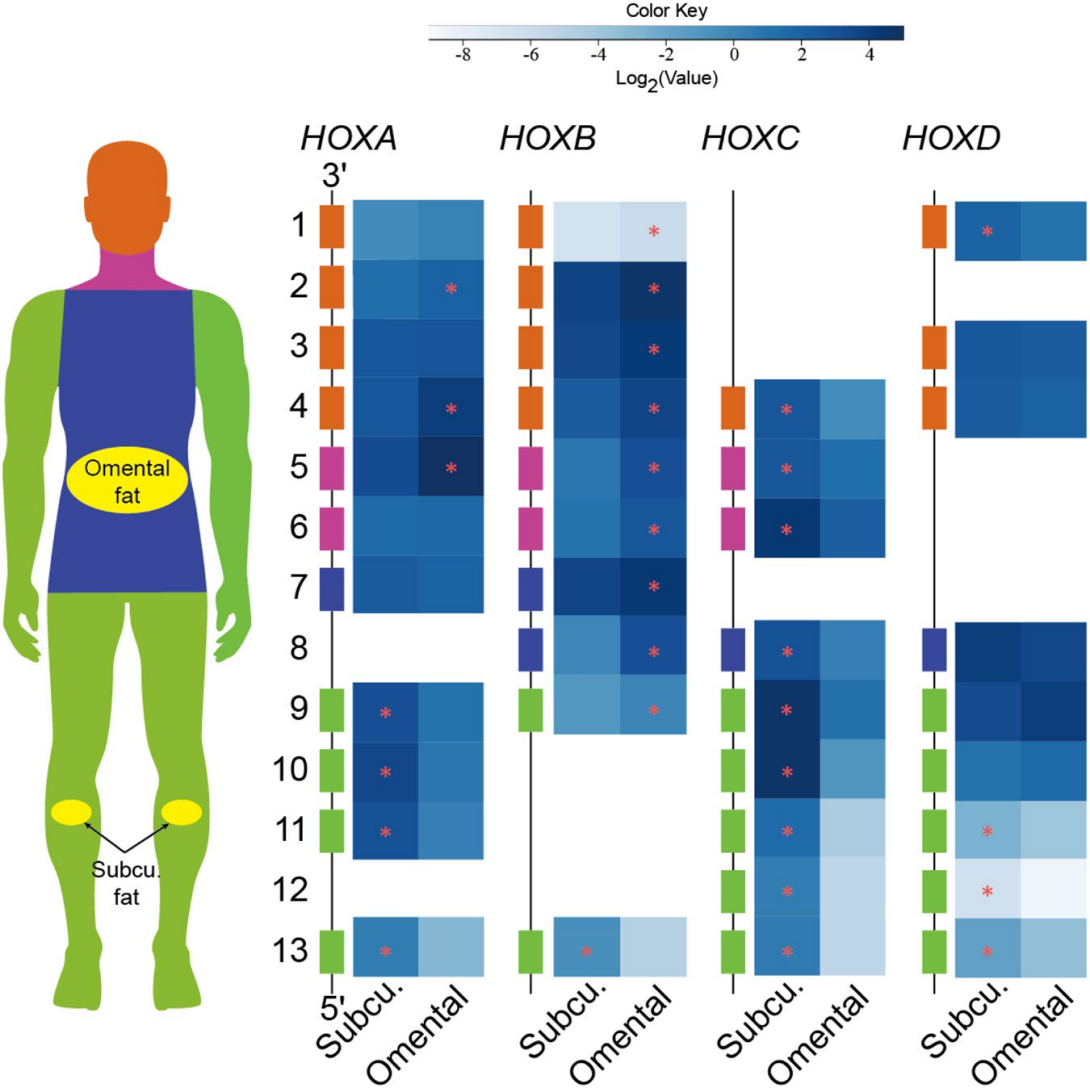
Heat map representation of expression patterns of human *HOX* clusters in subcutaneous and omental adipose tissues. The 39 *HOX* genes are arranged into four distinct chromosomal clusters and designated as *HOXA*, *HOXB*, *HOXC*, and *HOXD*. The locations of *HOX* gene expression in the human body are indicated with apricot, violet, blue, and green. The GTEx biospecimen source sites for collection of the following adipose tissues are highlighted with yellow: subcutaneous adipose tissues are derived from beneath the skin of the leg; and omental adipose tissues are collected from the large fold of parietal peritoneum. The scale bar depicts the degrees of log2 expression value (white, low expression; blue, high expression). The yellow stars indicate differential expression (DE) between adipose tissues (FDR < 0.01).

Considering differential expression of some development-related genes between adipose depots, expression pattern of homeotic genes in subcutaneous and omental adipose tissues were investigated. Interestingly, many homeobox (*HOX*) genes, a subfamily of homeotic genes, showed a distinct expression pattern in subcutaneous and omental adipose tissues (Fig. 6, Supplementary Table 6). First, the *HOXA* cluster displayed an anterior-to-posterior expression pattern. Specifically, expression levels of 3′ *HOXA4* gene controlling anterior regions and a central group gene, *HOXA5*, had a significantly higher expression in omental adipose tissue compared to subcutaneous adipose tissue; whereas, 5′ *HOXA* genes (*HOXA9, HOXA10*, *HOXA11*, and *HOXA13*) controlling posterior regions had a significantly higher expression in leg subcutaneous compared to omental adipose tissue. Second, *HOXB* and *HOXC* clusters showed an almost opposite expression pattern regardless of the group. The expression level of the *HOXB* cluster was significantly higher in omental adipose tissue than subcutaneous adipose tissue except for *HOXB13*; whereas, the *HOXC* cluster had a significantly greater expression in subcutaneous adipose tissue compared to omental adipose tissue. Last, the *HOXD* cluster showed a similar expression pattern as the *HOXC* cluster except for *HOXD9*, *HOXD10, and HOXD12*. In summary, the unique expression pattern of these four *HOX* clusters in subcutaneous and omental adipose tissue appears to be required for the development of human adipose depots.

## Discussion

Transcriptomic data from the GTEx project served as a valuable asset and have been used for studies on genetic variation and gene regulatory networks in multiple human tissues^24,33–35^. Due to large-scale sampling, these data enabled us to perform comprehensive profiling of tissue-specific genes. In this study, novel adipose-specific genes were identified using large-scale analysis of the human transcriptome, and their relation to obesity-related phenotypes was investigated through integrative analysis. Our approaches present effective identification of adipose-specific genes and an evidence base for a causal association between expression of adipose-specific genes with human obesity and related metabolic disorders.

In our study, 3 novel adipose-specific protein-coding genes (*CSN1S1*, *SLC19A3*, and *LVRN*) were identified through a comprehensive assessment with 44 non-adipose tissues. Their high expressions in either or both subcutaneous and omental adipose tissues suggest an unknown genetic mechanism underlying adipose tissue development and functions. Besides its nutritional role as a milk protein, CSN1S1 protein is also expressed in human blood monocytes where it induces the expression of proinflammatory cytokines such as IL-1β, IL-8, and IL-6 via toll-like receptor 4 (TLR4). Given these cytokines stimulate various innate and adaptive immune responses and play a key role in inflammatory and autoimmune disorders^30^ and an exclusive expression of *CSN1S1* in subcutaneous adipose tissue, a proinflammatory role of CSN1S1 protein in subcutaneous adipose tissue needs to be investigated. SLC19A3, also known as thiamine transporter 2 (THTR2), is one of the transporters of thiamine which is a member of the B family of vitamins, and upon uptake into intestinal epithelial cells, thiamine is converted into a coenzyme required for energy production from glucose metabolism^36^. As a growing amount of evidence highlights the role of deficiency of essential micronutrients such as thiamine and its derivatives in the progress of obesity^31,37^, this adipose-specific thiamine transporter gains special interest as a future therapeutic target. The *LVRN* gene encodes aminopeptidase Q (APQ/AQPEP) which is a cell surface protein expressed on human extravillous trophoblasts (EVTs) in the placenta. It hydrolyzes N-terminal amino acid of multiple peptide substrates expressed abundantly in the placenta such as angiotension III, kisspeptin-10, and endokinin C^32^. During placentation, LVRN plays a regulatory role in EVT migration to the uterus and its similarity in enzyme activity to an adipocyte-derived aminopeptidase was reported^32^, but its expression and functions in adipocytes have yet to be investigated.

In addition, genes showing adipose-enhanced expression, that have not been reported regarding their function in adipose tissue, included beta-defensin 132 (*DEFB132*), CD300 molecule like family member g (*CD300LG*), and glycogenin 2 (*GYG2*). The human DEFB132, also named HEL-75, is a secretory protein that is previously reported to be expressed highly in the human epididymis at both the transcription and translation levels^38^. DEFB132 showed dose- and time-dependent antibacterial activity without affecting fertilization indicating its main role in host defense^38^. CD300LG, also referred to as nepmucin, CLM-9, and TREM-4, is a type I membrane glycoprotein containing a mucin-like domain, and a single immunoglobulin (Ig) V-like domain. It is expressed in vascular endothelial cells, mediates lymphocyte rolling via its mucin-like domain, and promotes lymphocyte adhesion and migration across endothelial cells through the Ig domain. It has been reported that CD300LG binds various polar lipids^39^. GYG2 is one of the two self-glucosylating glycogenins that initiate the synthesis of glycogen, a storage form of a large amount of glucose as an energy source^40^. Unlike *GYG1* which is ubiquitously expressed, *GYG2* was highly expressed in adipose tissue with a lesser degree in the brain and a very low degree in skeletal muscle which is consistent with a previous report^40^. Although a dispensable role of *GYG2* in liver glycogen synthesis has been reported because of low expression of *GYG2* in the liver, its function in adipose tissue remains to be investigated, focusing on the regulatory role of *GYG2* in glucose and lipid metabolism in adipose tissue and an extended role in whole-body glucose homeostasis. Also, expression patterns of well-known adipose-specific genes, such as *FABP4* and *ITLN1* (omentin), were confirmed by this study as previously reported elsewhere^41,42^. Combined analysis of the GEO database^43^, a functional annotation and literature search, revealed novel adipose-specific/enhanced genes, and highlight their biological functions and pathways where dysregulation may lead to changes in adult and child adiposity, as shown in the case of expression of *SLC19A3* gene which is found to be significantly elevated in the child obese population.

Over the last decade, GWAS have documented numerous single nucleotide polymorphisms (SNPs) or variations at genetic loci associated with obesity-related phenotypes^25–29^. However, most GWAS loci should be interpreted with caution because of potential issues, such as difficulty in defining specific functional genes that are phenotypically- or pathologically-related, due to broad candidate chromosome regions for a particular genetic variation. In our study, adipose-specific genes identified using the GTEx RNA-Seq data were used to facilitate a comprehensive understanding of their association with GWAS phenotypes. Our integrative analysis showed that these adipose-specific genes are associated with obesity and related diseases including obesity, type 2 diabetes, blood lipid concentration, and inflammation. A robust association of inflammation-related GWAS candidate genes with omental adipose DEGs indicates omental adipose tissue plays a significantly more important role in inflammatory responses than subcutaneous adipose tissue. Further combined analyses on DEGs with GWAS revealed that some DEGs are perfectly matched with GWAS candidate genes for hip circumferences, waist circumferences, and waist-to-hip ratio. Since these body attributes are derived from regional fat deposition, our observation through the analyses is consistent with the notion that distribution of fat is regulated by genetic factors including differentially expressed genes in subcutaneous or visceral adipose tissue and genetic variants are linked to altered distribution of fat^44^. In this regard, the observed different expression levels of these genes between subcutaneous and omental adipose tissues may explain the positive relationship between these DEGs and distribution of adipose tissue depots in our study.

KEGG pathway analysis revealed that ligand-receptor interaction is one of the pathways that are enriched in relation to DEGs in subcutaneous adipose tissue. In this enriched pathway, genes encoding γ-aminobutyric acid type A receptor alpha 4 subunit (GABRA4), γ-aminobutyric acid type A receptor Pi subunit (GABRP), neuropeptide Y receptor Y2 (NPY2R), relaxin family peptide receptor 2 (RXFP2), and opioid receptor kappa 1 (OPRK1) are involved. In the brain, the γ-aminobutyric acid type A receptors (GABA_A_Rs), such as GABRA4 and GABRP are responsible for neurotransmitter signalling between NPY/AgRP neurons and POMC/CART neurons^45,46^, regulating food intake and body weight^47,48^. In adipocytes, the presence of γ-aminobutyric acid (GABA), GABA-synthesizing enzyme, GABA transporters, and GABA receptors suggests a functional role of GABA in adipose tissue^49^. Also, a very recent rodent study mentions the potential link of peripheral GABA signaling to the development of systemic metabolic dysfunction in obesity^50^. In this regard, those differentially expressed receptors in subcutaneous adipose tissue may play a significant role in peripheral adipose metabolism via systemic/paracrine signalling and needs to be further investigated. In addition, matrix metalloproteinases (MMPs)-mediated tissue remodelling was enriched with subcutaneous adipose DEGs encoding matrix metalloproteinase-3 (MMP-3) and -13 (MMP-13). This suggests that modification of structural integrity through these MMPs may be essential for distinct features of subcutaneous adipose tissue. MMPs are capable of cleaving protein components of extracellular matrix (ECM) and ensuing ECM remodeling plays important roles in maintaining adequate tissue function^51,52^. It has recently been reported that inhibition of MMP-13 led to reduced body adiposity in mice and decreased adipocyte differentiation of 3T3-L1 preadipocytes, suggesting an involvement of MMPs in adipocyte development^53^. Thus, these MMPs may play an important role in providing morphological and functional characteristics of subcutaneous adipose tissue as a protective tissue and further investigation is needed. On the other hand, most omental adipose DEGs were enriched in immune- and inflammation-related pathways including the JAK-STAT signaling pathway, IL-17 signaling pathway and TNF signaling pathway, implicating the role of their gene product in the regulation of immune/inflammatory responses as proinflammatory cytokines and chemokines. These data are consistent with previous evidence that adaptive immunity might be the most significant characteristic for omental adipose tissue^54^. In particular, some genes involved in immune/inflammatory responses and infection (e.g., *IL19*, *MAPK15*, and *SERPINB10*) and components of a complement system (e.g., *C4B*, *VTN*, *BDKRB1*, and *C4BPA*) have not been previously reported regarding their expression and functions in human omental adipose tissue. Therefore, these inflammation- or complement-related DEGs may need to be studied to fully characterize the immune system of omental adipose tissue.

*HOX* genes encode transcription factors that dominate the expression of developmental genes responsible for determining the specific anatomical structures, and regulate regional patterning of the body^55^. In humans, a total of 39 *HOX* gene family members are organized in four paralogous genomic clusters on 4 different chromosomes^56^. During body axis elongation, expression of the *HOX* genes controls a regional identity in a temporal and spatial collinear manner. In particular, 3′ *HOX* genes in group 1–4 (cervical) control the development of the branchial area, central *HOX* genes in groups 5–8 control the thoracic portion of the body, and 5′ *HOX* genes in groups 9–13 control the lumbo-sacral region^57^. Given these roles of *HOX* genes, our transcriptional profiling data suggest that the *HOXA* cluster displays a unique expression in regional fat depots following the anterior–posterior axis. Also, as shown in Fig. 5, different expression patterns among the *HOXA*, *HOXB*, *HOXC*, and *HOXD* clusters were observed between subcutaneous and omental adipose tissues. The significantly greater expression of *HOXC* cluster and some of *HOXD* genes in subcutaneous adipose tissue suggests that these genes may be important for controlling the development of subcutaneous adipose tissue. On the contrary, the majority of *HOXB* genes had a significantly greater expression in omental adipose tissue, suggesting that *HOXB* cluster genes may play critical roles in characterization of omental adipose tissue. Taken together, it appears that different *HOX* codes, which are combinatorial expression patterns of *HOX* genes for the specification of regional identity^58^, exist between subcutaneous and omental adipose tissues. These depot-specific *HOX* codes might partially contribute in differentiating the anatomical structure, regulating distribution, and determining functional characteristics of the two adipose depots.

Comprehensive analysis of the relationship between adipose-specific genes and genetic loci associated with obesity traits is essential to elucidating the genetic architecture underlying human obesity and related diseases. Thus, our findings on novel adipose-specific genes provide new insights into the susceptibility genes of obesity and related diseases. Continued attempts in characterization of adipose-specific genes and DEGs will be needed to increase our understanding of the etiology of obesity. In conclusion, our analysis identified adipose-specific genes and revealed evidence for their relationship with genetic causality of obesity, providing an important foundation for further elucidation of genetic factors and therapeutic target genes of obesity and associated diseases.

## Methods

### Data Collection

The most recent publicly available RNA-seq data reported in the GTEx Analysis V7 (dbGaP Accession: phs000424.v7.p2, release date: June 30, 2017) release was downloaded from the GTEx portal (www.gtexportal.org). This comprehensive data set includes gene expression values, which were normalized using transcripts per million (TPM), from non-diseased normal tissues of postmortem human donors (n = 752). For the current study, samples with RNA integrity number (RIN) of 6.0 or higher were initially chosen. Mammary tissue was excluded from the data due to its high fat content, as well as non-tissue samples such as cultured transformed fibroblasts and EBV-transformed lymphocytes. Our analysis was then confined to tissues from at least 30 donors, resulting in a total of 10,098 sampled tissues from 46 tissues including subcutaneous and omental adipose tissues, 13 brain subregions, whole blood, and other organ tissues as listed in Supplementary Table 1. The sample size for each tissue ranged from 35 to 560.

### Data Analysis

Initially, 56,202 human protein-coding and noncoding genes were filtered by criteria based on expression values (i.e., in a given tissue, at least 20% of TPM values is more than 0.1 and median TPM > 0.5). After filtering, adipose-specific genes and adipose-enhanced genes were identified using a median fold change with a concomitant one-way ANOVA-based test and the Benjamini–Hochberg false discovery rate (FDR) adjustment for multiple testing using the Bioconductor limma package^59^. Adipose-specific genes were defined as genes with a median fold change higher than 5.0 in adipose tissue *versus* all other tissues and FDR-adjusted *P* value < 0.01 in all pair-wise comparisons between adipose tissue and the rest of tissues. In detail, genes with at least a median 5-fold change and whose expression is significantly different between tissues based on ANOVA based test, formulated as [FDR of *i versus j* pairwise comparison] < 0.01, where *i* = A1 or A2 (adipose) and *j* = B, C, D, ···, or AS (non-adipose), were selected. Adipose-enhanced genes were defined as genes having a median fold change higher than 5.0 in adipose tissue *versus* all other tissues, with an exception of 1 tissue or 2 other tissues (for those 1 or 2 tissues, higher median expression in adipose tissue was selected) and FDR-adjusted *P* value < 0.01. For example, an adipose-enhanced *CD300LG* gene showed 26% median expression level in testis compared to subcutaneous adipose tissue, which is less than 5-fold higher, but 3.8-fold higher, in subcutaneous adipose tissue (Supplementary Table 1). Genes showing higher expression in those 1 or 2 tissues than in adipose tissue were excluded from the category of adipose-enhanced. For grouping protein-coding and noncoding genes [long intergenic non-coding RNA (linc RNA), pseudogene, small nuclear RNA (snRNA), and antisense RNA], the Ensembl Gene IDs were aligned to the human reference genome using the Ensembl gene GRCh38 release 91 annotation file (https://www.ensembl.org/info/data/ftp/index.html). Heat maps were generated using the R package heatmap3^60^.

### Semi-quantitative RT-PCR

Total RNAs from the adult human brain, heart, kidney, lung, liver and skeletal muscle were purchased from Agilent Technologies (Santa Clara, CA) and adult human RNA from adipose tissue was purchased from Clontech Laboratories (Mountain View, CA). To measure the quantity of RNA, a Nanodrop spectrophotometer (Thermo Scientific, Wilmington, DE) was used. Approximately 1 µg of RNA was reverse-transcribed in a 20 µL total reaction to cDNA using Moloney murine leukemia virus (M-MLV) reverse transcriptase (Invitrogen). The thermal cycle of reverse transcription (RT) was 65 °C for 5 min, 37 °C for 52 min, and 70 °C for 15 min. Exactly 1 µL of cDNA samples was used as a template for PCR in a 25 µL total reaction with AmpliTaq Gold DNA polymerase (Applied Biosystems, Carlsbad, CA). The conditions for this reaction were 95 °C for 10 min, appropriate cycles with linear amplification ranges of 94 °C for 30 s, 55 °C for 30 s, 72 °C for 30 s, with an additional extension step at 72 °C for 10 min. PCR products were separated by using 1% agarose gel electrophoresis. Following forward and reverse primers for humans were designed on different exons for multi-exon genes to avoid genomic DNA contamination: *CSN1S1* (forward: 5′-CCTACCCCTAT GCTGTTT-3′, reverse: 5′-TCCTTGAGAGGAGAAATTCA-3′), *SLC19A3* (forward: 5′-ACTTGCCA GTCAGCATT-3′, reverse: 5′-GTTTGTTGCGATGAGGTTA-3′), and *LVRN* (*AQPEP*) (forward: 5′-GGAGAACCGTAACTACAGATTT-3′, reverse: 5′-TGAAAGATAGCCACAAGCTAT-3′). Human cyclophilin (*CYC*; forward: 5′-CTCCTTTGAGCTGTTTGCAG-3′, reverse: 5′-CACCACATGCTTG CCATCC-3′) was used as a reference gene.

### Western blot analysis

Human tissue lysates from subcutaneous adipose tissue, brain, heart, kidney, lung, liver and skeletal muscle were purchased from Protein Biotechnologies Inc (Ramona, CA). Western blot analysis with tissue protein lysates was performed as described in our previous report^61^. In detail, equal amounts of protein lysates were loaded onto gels before wet-transfer to PVDF membranes (Bio-Rad, Hercules, CA). The membranes were blocked for 30 min and then incubated with CSN1S1 (H00001446-D01P; 1:750; Novus Biologicals, LLC, Centennial, CO) or SLC19A3 (NBP1–69703; 1:500; Novus Biologicals, LLC) antibodies at 4 °C overnight. The next day, after washing, an appropriate secondary antibody (HRP-linked anti-rabbit IgG (HAF008); 1:5000; R&D systems Inc., Minneapolis, MN) was applied to the membrane before washing and developing with ECL plus reagents and X-ray films (both materials from GE Healthcare Biosciences, Pittsburgh, PA). GAPDH (antibody: 10494-1-AP; 1:1000; Proteintech Group, Chicago, IL) was used as a reference protein.

### Profiling of adipose-specific genes

To investigate biological functions and subcellular localization of three unreported common adipose-specific genes, the GeneCards database of human genes (http://www.genecards.org/) and literature were explored. The PubMed database was searched for previous studies using the keywords ‘gene name AND adipose’ or ‘gene name AND adipocyte’ or ‘gene name AND obesity’ and functionally unreported genes in adipose tissue were determined. For further identification of obesity relatedness of each of those genes, data were retrieved from the Gene Expression Omnibus (GEO) Series (GSE) through the GEO2R interface (http://www.ncbi.nlm.nih.gov/geo/geo2r/).

### Comprehensive comparison between adipose depots

Read count matrix from RNA-Seq of subcutaneous adipose tissue and omental adipose tissue was downloaded from GTEx portal. The Bioconductor DESeq. 2 package^62^ was used to normalize all samples for sequencing depth and calculate the DEGs between subcutaneous adipose tissue and omental adipose tissue. To obtain significant DEGs, the combined criteria of FDR-adjusted *P* value < 0.01 and the absolute log2 fold change >3 was used, where a fold change is defined as the expression in samples of subcutaneous adipose tissue divided by the expression in samples of omental adipose tissue.

### Association analyses with GWAS phenotypes

The GWAS data used for our analyses were obtained from the public GWAS Catalog (https://www.ebi.ac.uk/gwas/) on 06/25/2018. Reported and mapped genes of GWAS shared with adipose-specific/enhanced genes and DEGs were used to analyze the relationships between gene expression and phenotypic traits in adipose tissues. Whole genome visualization was performed by PhenoGram^63^ to plot DEGs with associated traits using categories of GWAS phenotypes.

### GO functional and pathway enrichment analyses

For the sets of DEGs, the Gene Ontology (GO) function and Kyoto Encyclopedia of Genes and Genomes (KEGG) pathway enrichment analyses were performed using R package, clusterProfiler (version 3.6.0)^64^. The enriched GO terms and KEGG pathways were displayed with the significance of the enrichment of each gene set under the criteria of FDR-corrected *P* value < 0.05. The visualization of relevant KEGG pathways was generated using GOPlot (version 1.0.2)^65^.

## Supplementary information

Supplementary Figures

Supplementary Table 1

Supplementary Table 2

Supplementary Table 3

Supplementary Table 4

Supplementary Table 5

Supplementary Table 6
