# Supplementary material for: Integrative Analysis Revealing Human Adipose-Specific Genes and Consolidating Obesity Loci
Source: Sci Rep. 2019 Feb 28;9:3087. doi: 10.1038/s41598-019-39582-8 (PMC6395763; doi:10.1038/s41598-019-39582-8)
Supplement: Supplementary file 1 — Supplementary Figures [file 41598_2019_39582_MOESM1_ESM.pdf]

Integrative Analysis Revealing Human Adipose-Specific Genes and Consolidating Obesity Loci

Jinsoo Ahn[1,†], Huiguang Wu[1,2,†], and Kichoon Lee[1,*]

[1] Department of Animal Sciences, The Ohio State University, Columbus, OH, 43210, USA
[2] College of Veterinary Medicine, Yangzhou University, Yangzhou 225009, China.
[*] Corresponding author: lee.2626@osu.edu
[†] These authors contributed equally to this work

Supplementary Figure 1. Expression of *SLC19A3* in subcutaneous adipose tissue (*Far left*) compared to 43 non-adipose tissues.

Supplementary Figure 2. Expression of *SLC19A3* in omental adipose tissue (*Far left*) compared to 43 non-adipose tissues.

Supplementary Figure 3. Expression of *CSN1S1* in subcutaneous adipose tissue (*Far left*) compared to 43 non-adipose tissues.

Supplementary Figure 4. Expression of *LVRN* (*AQPEP*) in subcutaneous adipose tissue (*Far left*) compared to 43 non-adipose tissues.

Supplementary Figure 5. Global expression pattern of 26,200 genes (ranked by the expression level in subcutaneous adipose tissue after removing duplicates and zero values from 56,202 genes). Far left: subcutaneous adipose tissue; 5th from the right: Testis (showing expression of the largest number of enriched genes as reported previously (Uhlén et al., 2015); Rest: additional 43 non-adipose tissues.

Supplementary Figure 6. Global expression pattern of 27,543 genes (ranked by the expression level in omental adipose tissue after removing duplicates and zero values from 56,202 genes). Far left: subcutaneous adipose tissue; 5th from the right: Testis (showing expression of the largest number of enriched genes as reported previously (Uhlén et al., 2015); Rest: additional 43 non-adipose tissues.

Supplementary Figure 7. Representative light (*left*) and dark (*right*) images of RT-PCR and/or Western blot analysis of CSN1S1, SLC19A3 and LVRN (AQPEP). CYC and GAPDH were used as reference genes. Subcutaneous adipose tissue (Ad), brain (Br), heart (He), kidney (Ki), lung (Lu), liver (Li), and skeletal muscle (Mu).

Supplementary Figure 8. Volcano plot representation of DEGs between adipose depots. Differential expression analysis for 37,167 genes with available adjusted p-values was applied to the comparison of mean expression values between subcutaneous and omental adipose tissues, using the DESeq2 package. The negative log10-transformed FDR adjusted p-values are plotted against the log ratios (log2 fold change) between subcutaneous and omental adipose tissues. Green dots represent differentially expressed genes that show both at least 8-fold changes and high statistical significance (FDR <0.01). Black dots represent all other genes.

Supplementary Table 1
Subcutaneous adipose-specific/enhanced genes which are functionally unreported in adipose tissue
Omental adipose-specific/enhanced genes which are functionally unreported in adipose tissue
Subcutaneous adipose-specific/enhanced genes
Omental adipose-specific/enhanced genes
Tissue sample

Supplementary Table 2
Adipose-specific/enhanced genes associated with four categories of GWAS phenotypes
List of four categories of GWAS phenotypes

Supplementary Table 3
Differentially expressed genes between subcutaneous and omental adipose tissue

Supplementary Table 4
DEGs in subcutaneous and omental adipose tissue & associated seven categories of GWAS phenotypes
List of seven categories of GWAS phenotypes

Supplementary Table 5
KEGG Enrichment of upregulated DEGs in subcutaneous adipose tissue
KEGG Enrichment of upregulated DEGs in omental adipose tissue

Supplementary Table 6
Expression of *Hox* genes in subcutaneous and omental adipose tissue

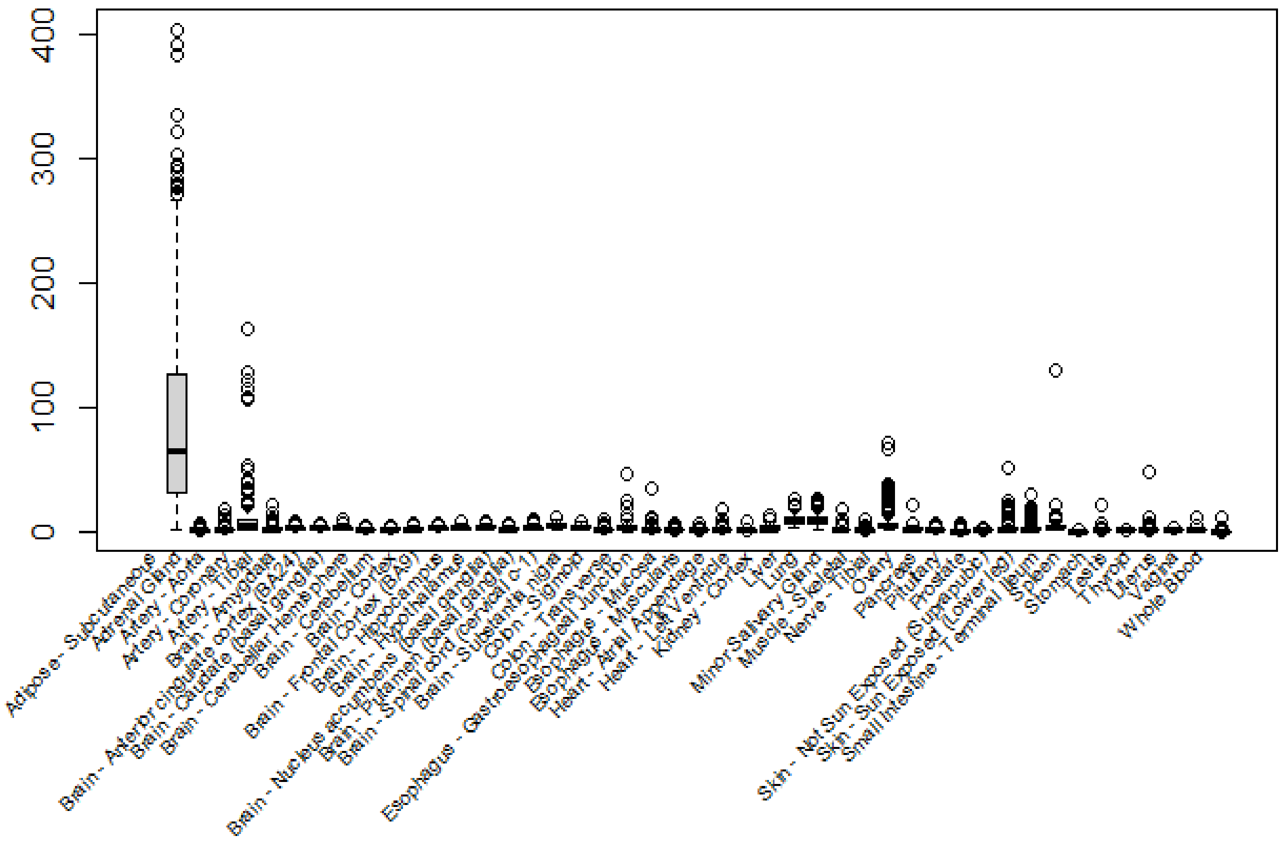

Supplementary Figure 1. Expression of *SLC19A3* in subcutaneous adipose tissue (*Far left*) compared to 43 non-adipose tissues.

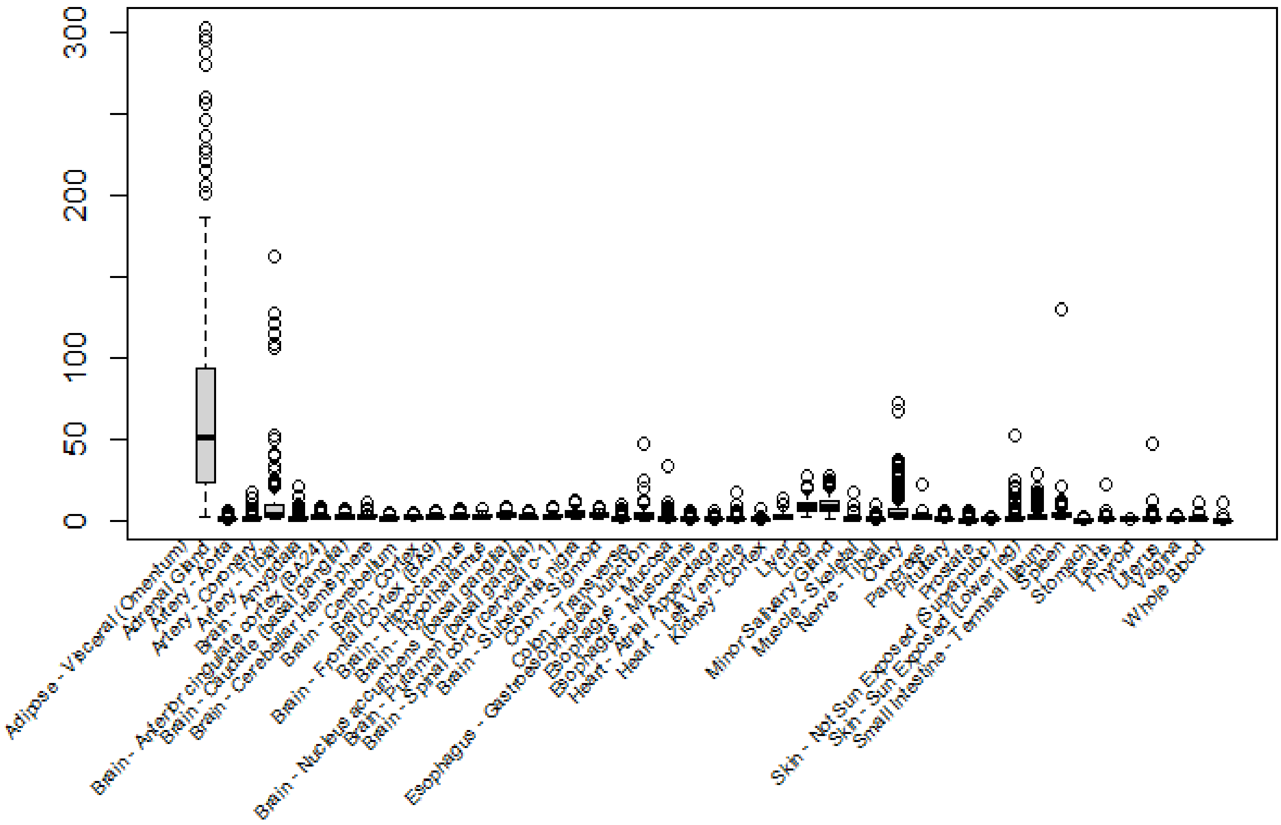

Supplementary Figure 2. Expression of *SLC19A3* in omental adipose tissue (*Far left*) compared to 43 non-adipose tissues.

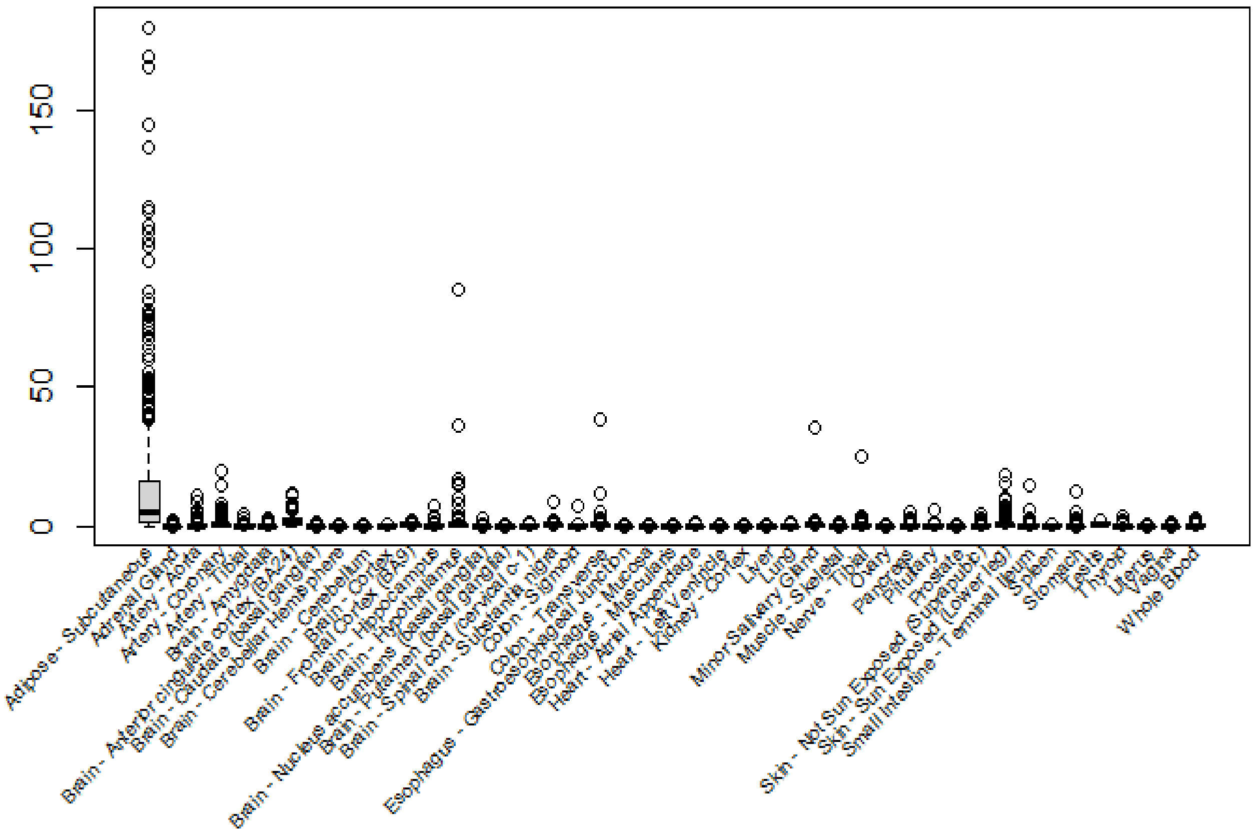

Supplementary Figure 3. Expression of *CSN1S1* in subcutaneous adipose tissue (*Far left*) compared to 43 non-adipose tissues.

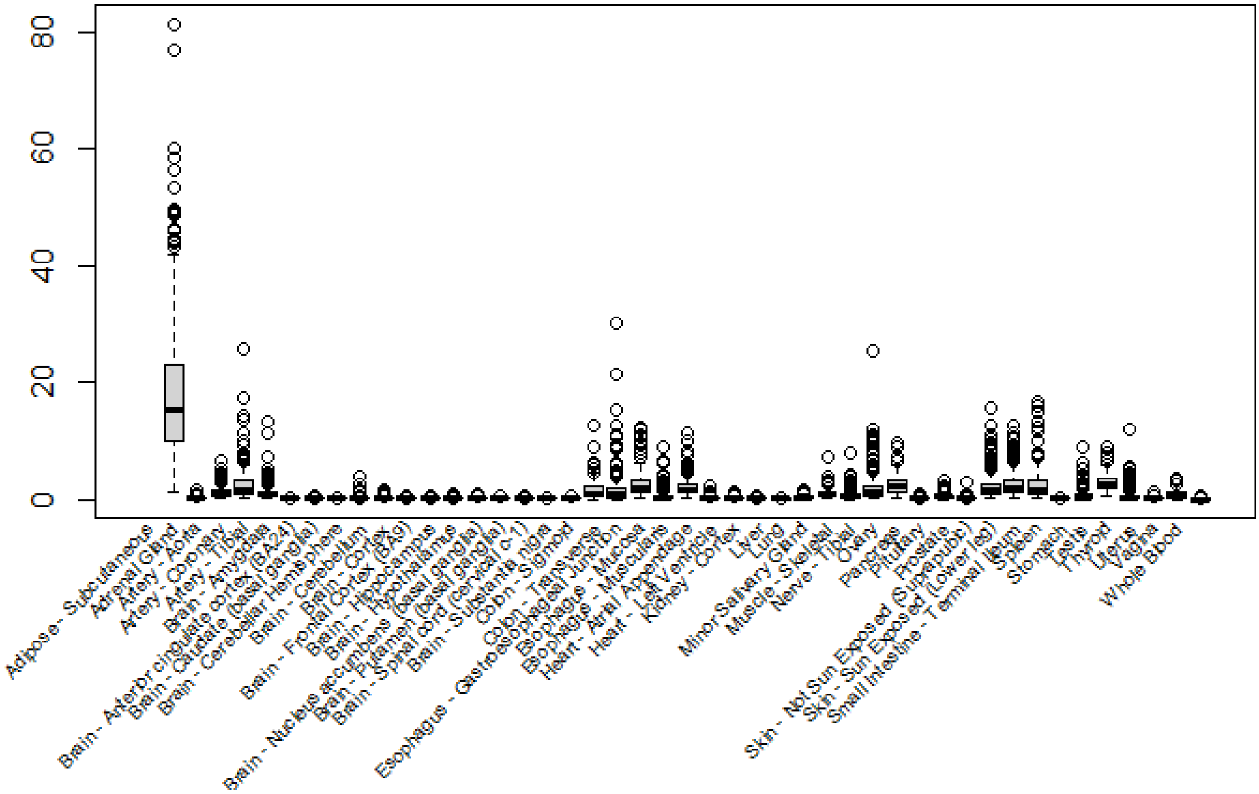

Supplementary Figure 4. Expression of *LVRN* (*AQPEP*) in subcutaneous adipose tissue (*Far left*) compared to 43 non-adipose tissues.

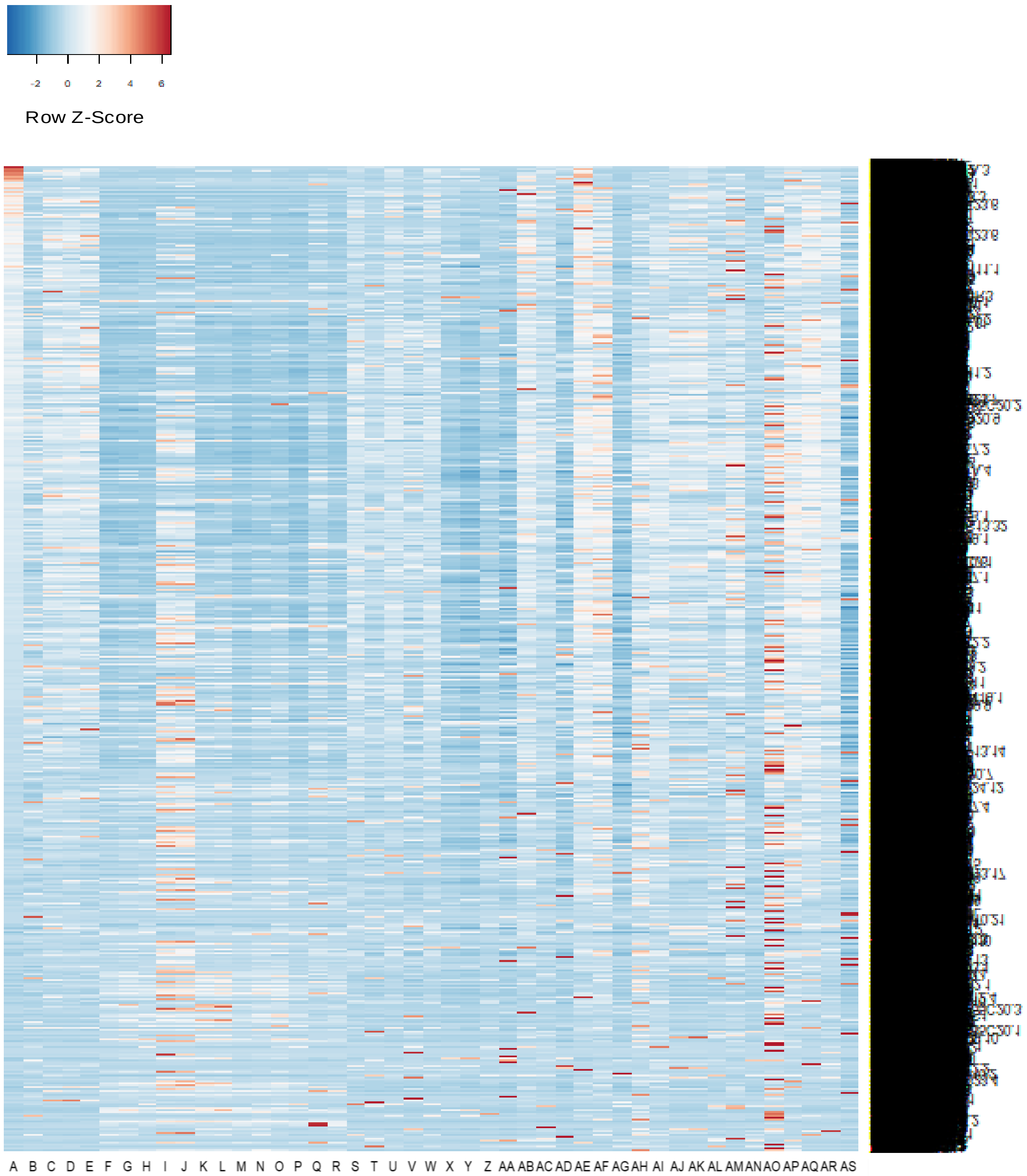

Supplementary Figure 5. Global expression pattern of 26,200 genes (ranked by the expression level in subcutaneous adipose tissue after removing duplicates and zero values from 56,202 genes). Far left: subcutaneous adipose tissue; 5th from the right: Testis (showing expression of the largest number of enriched genes as reported previously (Uhlén et al., 2015); Rest: additional 43 non-adipose tissues.

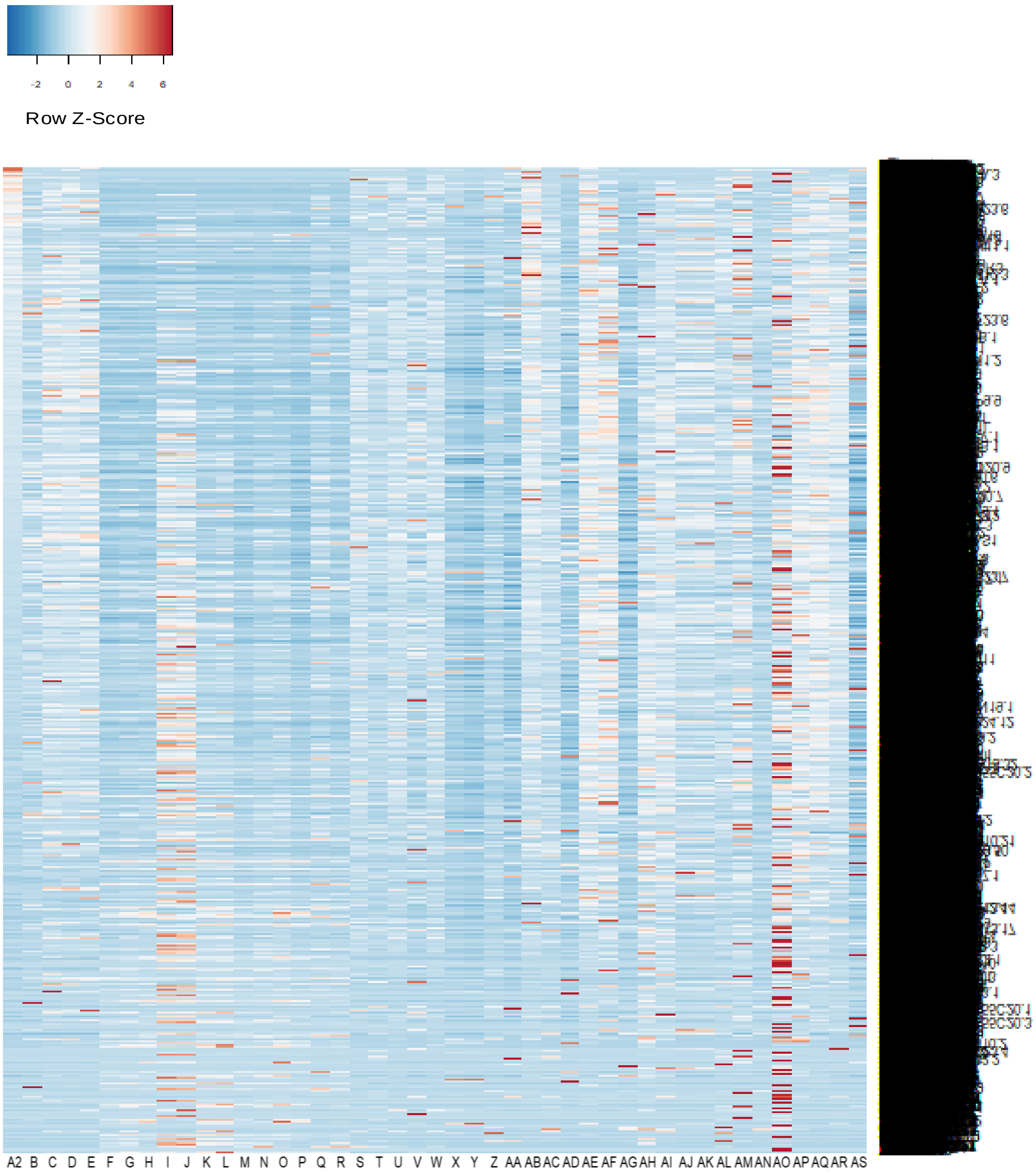

Supplementary Figure 6. Global expression pattern of 27,543 genes (ranked by the expression level in omental adipose tissue after removing duplicates and zero values from 56,202 genes). Far left: subcutaneous adipose tissue; 5th from the right: Testis (showing expression of the largest number of enriched genes as reported previously (Uhlén et al., 2015); Rest: additional 43 non-adipose tissues.

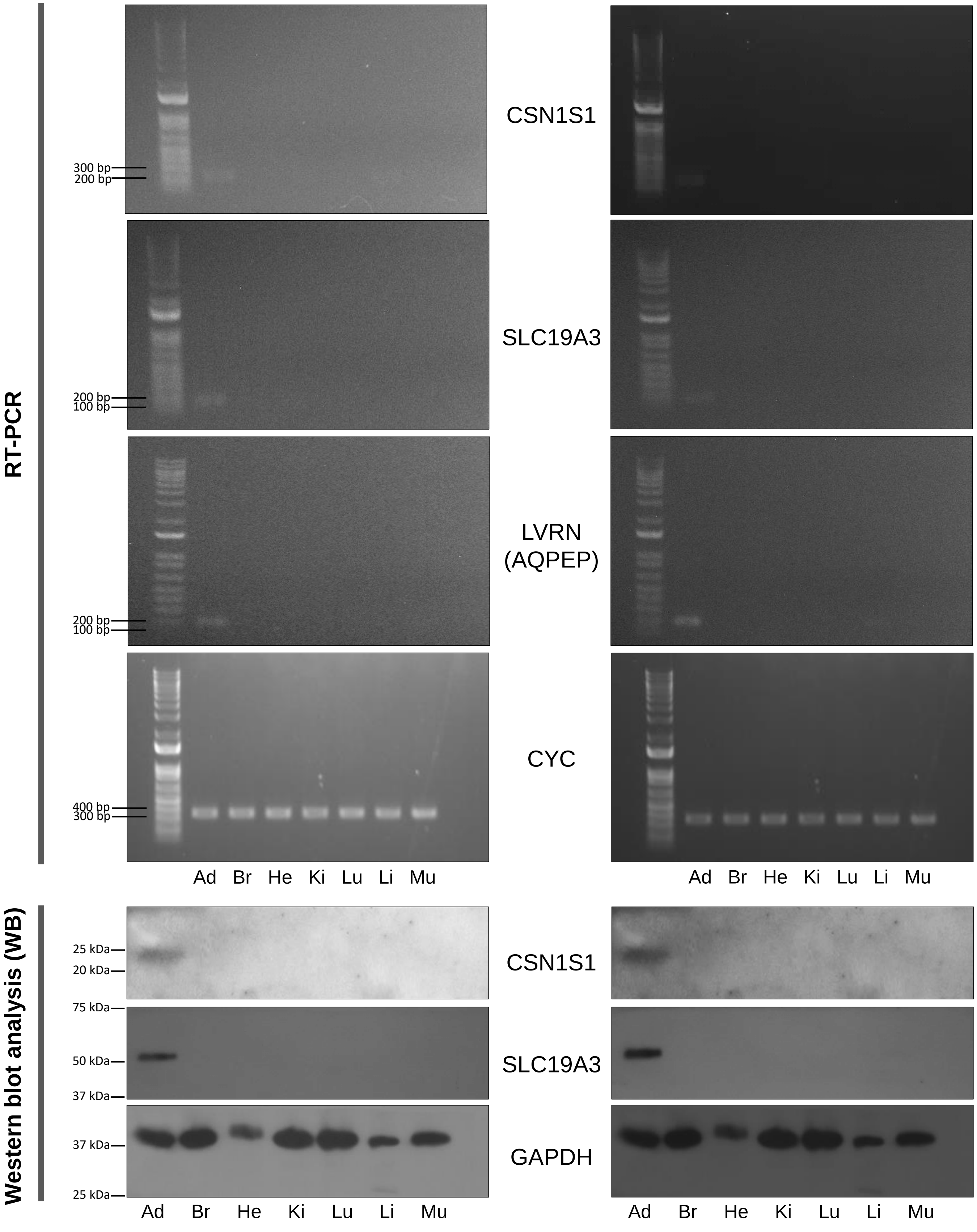

Supplementary Figure 7. Representative light (*left*) and dark (*right*) images of RT-PCR and/or Western blot analysis of CSN1S1, SLC19A3 and LVRN (AQPEP). CYC and GAPDH were used as reference genes. Subcutaneous adipose tissue (Ad), brain (Br), heart (He), kidney (Ki), lung (Lu), liver (Li), and skeletal muscle (Mu).

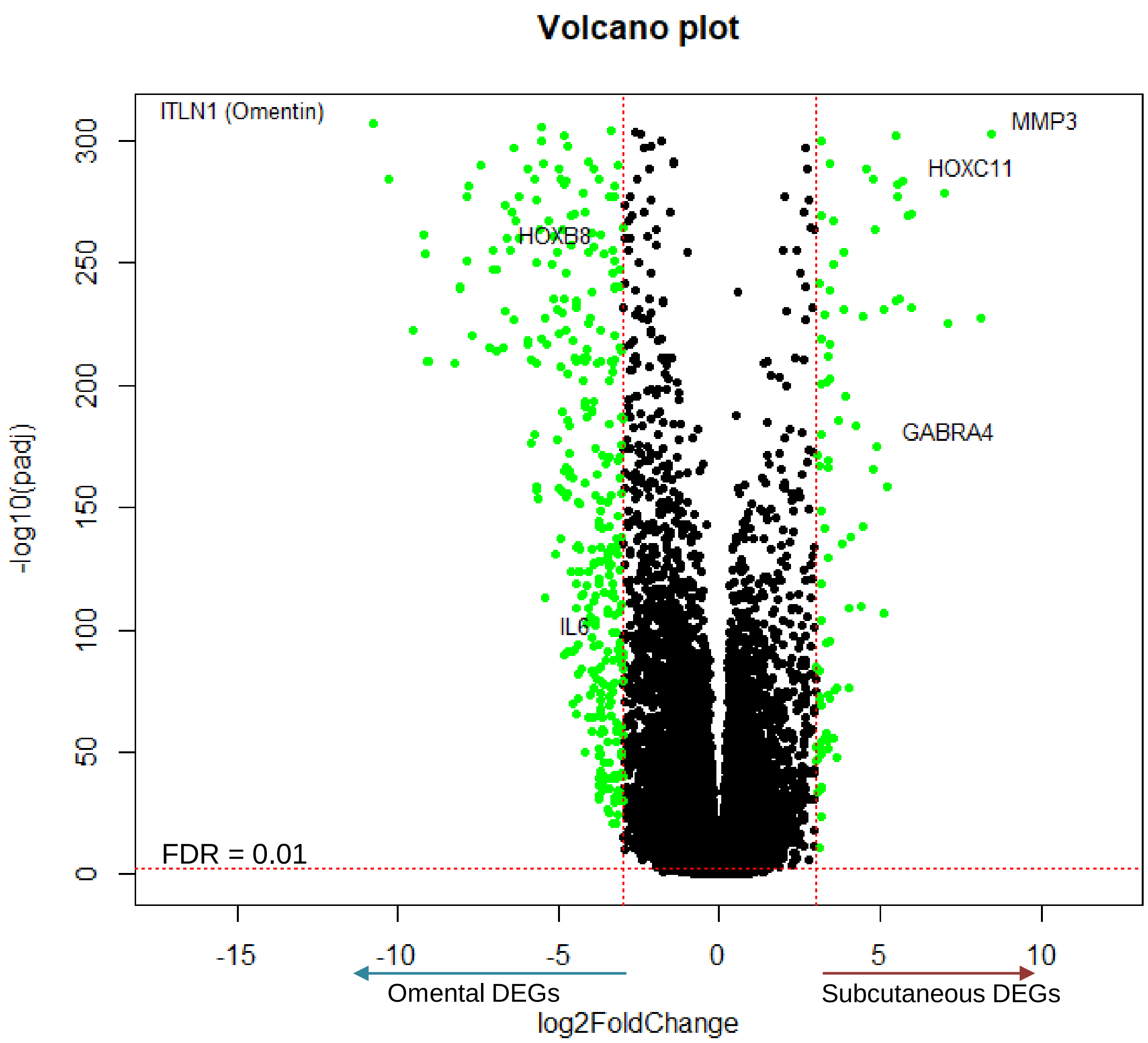

Supplementary Figure 8. Volcano plot representation of DEGs between adipose depots. Differential expression analysis for 37,167 genes with available adjusted p-values was applied to the comparison of mean expression values between subcutaneous and omental adipose tissues, using the DESeq2 package. The negative log10-transformed FDR adjusted p-values are plotted against the log ratios (log2 fold change) between subcutaneous and omental adipose tissues. Green dots represent differentially expressed genes that show both at least 8-fold changes and high statistical significance (FDR <0.01). Black dots represent all other genes.
